# Urban Community Gardeners' Knowledge and Perceptions of Soil Contaminant Risks

**DOI:** 10.1371/journal.pone.0087913

**Published:** 2014-02-06

**Authors:** Brent F. Kim, Melissa N. Poulsen, Jared D. Margulies, Katie L. Dix, Anne M. Palmer, Keeve E. Nachman

**Affiliations:** 1 Johns Hopkins Center for a Livable Future, Baltimore, Maryland, United States of America; 2 CLF-Lerner Fellow, Johns Hopkins Center for a Livable Future, Baltimore, Maryland, United States of America; 3 Department of Environmental Health Sciences, Johns Hopkins Bloomberg School of Public Health, Baltimore, Maryland, United States of America; 4 Department of International Health, Johns Hopkins Bloomberg School of Public Health, Baltimore, Maryland, United States of America; 5 Department of Geography and Environmental Systems, University of Maryland, Baltimore, Maryland, United States of America; 6 Community Greening Resource Network, Parks & People Foundation, Baltimore, Maryland, United States of America; 7 Department of Health, Behavior and Society, Johns Hopkins Bloomberg School of Public Health, Baltimore, Maryland, United States of America; 8 Department of Health Policy and Management, Johns Hopkins Bloomberg School of Public Health, Baltimore, Maryland, United States of America; Kansas State University, United States of America

## Abstract

Although urban community gardening can offer health, social, environmental, and economic benefits, these benefits must be weighed against the potential health risks stemming from exposure to contaminants such as heavy metals and organic chemicals that may be present in urban soils. Individuals who garden at or eat food grown in contaminated urban garden sites may be at risk of exposure to such contaminants. Gardeners may be unaware of these risks and how to manage them. We used a mixed quantitative/qualitative research approach to characterize urban community gardeners' knowledge and perceptions of risks related to soil contaminant exposure. We conducted surveys with 70 gardeners from 15 community gardens in Baltimore, Maryland, and semi-structured interviews with 18 key informants knowledgeable about community gardening and soil contamination in Baltimore. We identified a range of factors, challenges, and needs related to Baltimore community gardeners' perceptions of risk related to soil contamination, including low levels of concern and inconsistent levels of knowledge about heavy metal and organic chemical contaminants, barriers to investigating a garden site's history and conducting soil tests, limited knowledge of best practices for reducing exposure, and a need for clear and concise information on how best to prevent and manage soil contamination. Key informants discussed various strategies for developing and disseminating educational materials to gardeners. For some challenges, such as barriers to conducting site history and soil tests, some informants recommended city-wide interventions that bypass the need for gardener knowledge altogether.

## Background

Urban community gardens—gardens tended by multiple households in an urban neighborhood—may offer a range of benefits. Studies have observed associations between community gardening and health [1]–[8], social [6], [9], and economic benefits [6], [7], [10], and gardening in general has been associated with cardiovascular [11], [12] and mental [13]–[15] health benefits. Historically, backyard and community gardens have made substantial contributions to the food supply; World War II “Victory Gardens” have been credited with providing an estimated 40% of the U.S. vegetable supply [16]. In urban settings, community gardens—and urban green spaces in general—may confer an additional set of social benefits [17]–[20] and ecosystem services [21], [22]. Urban green spaces also provide educational opportunities for urban residents, for whom parks and gardens may be their primary source of experience, knowledge, and valuation of nature.

Gardening in urban settings may also present health risks, including those stemming from exposure to contaminants such as heavy metals, organic chemicals, and asbestos that may be present in urban soils. Urban soils are often close to pollution sources, such as industrial areas and heavily trafficked roads. As a result, many soil contaminants have been found at higher concentrations with increasing proximity to urban centers [23]. In Baltimore, Maryland, prior soil analyses (Table S1 in File S1) have revealed high concentrations of lead at some sites [24]–[28], reflecting the city's long history of industrial activity, incinerators, and vehicular traffic, and raising concerns about lead exposure [24]. Table S2 in File S1 summarizes some of the more common urban soil contaminants, their sources, and health effects associated with exposure.

Gardeners can be exposed to contaminants by inadvertently ingesting soil, inhaling soil particles, or via dermal contact. Soil ingestion is of particular concern among children, who may ingest larger quantities of soil than adults (e.g., by putting their hands in their mouths), absorb higher levels of certain contaminants into their bloodstream [29], and are generally more sensitive to their effects. People who consume produce grown in contaminated environments also risk ingesting soil particles on the surfaces of plants [30]. Contaminants in soils including lead [31], cadmium [32], and arsenic [33] may accumulate in the tissues of vegetables grown in contaminated soils, posing another potential route of ingestion.

Urban gardeners may be unaware of how to manage these risks. Harms et al. [34], [35] surveyed 121 urban farmers and gardeners from Kansas, Indiana, and Washington, most of whom indicated they do not have sufficient knowledge of how to minimize health risks associated with gardening in contaminated environments and want more information on soil testing and best management practices. Gardeners may also benefit from information on soil remediation, i.e. removing, destroying, detoxifying, immobilizing or containing soil contaminants [36].

The purpose of our study is to characterize urban community gardeners' knowledge of risks associated with contaminated garden soils, their perceptions of these risks, their knowledge of how to assess and reduce these risks, the sources they draw upon for information on soil contamination, and the information and training needs they have related to soil contamination.

## Methods

To characterize urban community gardeners' knowledge and perceptions of soil contamination risks, we conducted surveys among urban community gardeners and semi-structured interviews with key informants in the gardening community.

### Gardener surveys

We conducted brief verbal surveys in-person or by phone with Baltimore community gardeners. The survey included questions regarding demographics, garden site history, and knowledge, perceptions, and practices related to soil contamination. To be eligible to participate, gardeners had to be at least 18 years of age and have been gardening at their current site for at least 6 months.

We partnered with the Community Greening Resource Network (CGRN) to identify gardens from which to recruit survey participants. CGRN is Baltimore's gardening support network and maintains a registry of community gardens in the Baltimore metropolitan area. We randomly selected 30 gardens from the CGRN registry of 70 food-producing community gardens, contacting leaders at the selected gardens to identify opportunities to survey gardeners. After experiencing difficulty reaching some garden leaders, we included additional gardens – recommended to us by representatives in the gardening community or identified through personal contacts – whose leaders were willing to help us arrange surveys.

As an incentive for participating gardeners, we collected soil samples from represented community gardens, sent the samples for analysis at the U.S. Department of Agriculture (USDA) soils lab, shared the results with garden leaders, and offered guidance in interpreting the results.

### Key informant interviews

We conducted semi-structured interviews with 18 purposively selected informants knowledgeable about community gardening and soil contamination in Baltimore City: representatives from City government urban agriculture-related programs (4), federal agency employees (2), a representative of a Baltimore community gardening organization (1), agricultural extension employees (2), Master Gardeners - trained volunteers who advise and educate the public on gardening (2), community garden leaders (4), and urban farmers (3). We distinguish farming from gardening by the intent to produce goods for sale.

Interviews focused on informants' perceptions of community gardeners' concerns about soil contamination, barriers to soil testing, and information needs related to soil contamination. When applicable, informants were also asked relevant questions about their roles and perspectives related to their employment in city, state, or federal agencies.

To identify major themes in the qualitative data, three members of the research team first developed a codebook that was organized by axial codes and sub-codes. Two researchers coded each transcript using Atlas/ti (v7); when discrepancies arose, we included all quotes assigned to a particular code by either researcher. We then generated reports of the text assigned to each code, writing reflective memos and pulling out illustrative quotes for each theme.

### Ethical considerations

The Johns Hopkins School of Public Health Institutional Review Board (IRB) approved this study. Study participants provided verbal informed consent prior to participating in surveys and interviews. Oral consent was deemed adequate by the IRB, eliminating the need to record identifying information in study documents. An IRB-approved oral consent script was read by trained investigators to study participants. A dated questionnaire served as a record that the oral consent process had been completed.

## Results

### Gardener demographics

**Figure 1:**
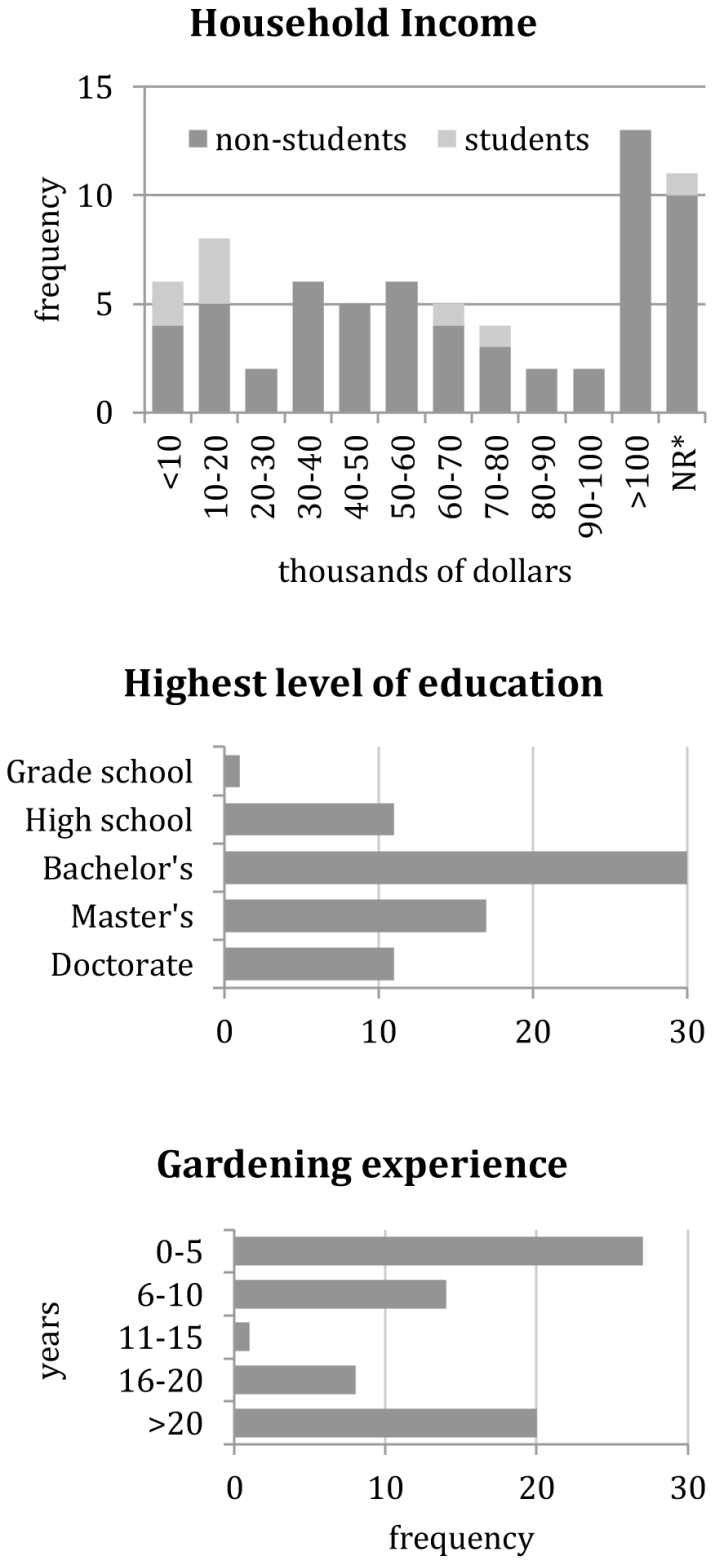
Additional gardener demographic information. * NR  =  No response.

Seventy gardeners, representing 15 community gardens from a range of socioeconomic census tracts, responded to our survey. Most were female (66%), lived within a quarter-mile of their garden plots (76%), and had been at their current gardens for less than four years (76%). The median age of surveyed gardeners was 45. See Figure 1 for additional gardener demographic information.

### Gardening practices and harvest use

Most (73%) surveyed gardeners indicated they avoid using commercial pesticides and fertilizers, relying instead on practices such as composting and mulching to promote soil fertility and suppress pests. Others (19%) reported using one or more commercial fertilizers and/or insecticides, several of which are allowed for use under USDA organic standards. Interview informants indicated that Baltimore's community gardeners generally garden without chemical inputs.

Among surveyed gardeners, 86% grew produce for home consumption, while the other 14% grew produce primarily for soup kitchens and other charitable uses. Among gardeners who grew produce for home consumption, almost half (45%) supplied over 60% of their family's produce intake from their community garden during the growing season.

### General knowledge and concerns about contaminants

To assess their knowledge about chemical contaminants, surveyed gardeners were asked to list the soil contaminants they are aware of (Table 1). Most (66%) gardeners mentioned lead, and to a lesser extent, other trace elements (19%) and some types of organic chemicals (36%).

**Table 1:** Open-ended responses to questions about soil contaminant knowledge: “What soil contaminants are you aware of that urban gardeners should be concerned about in general?” and “As a community gardener, do you have any concerns about hazards to your health?”

Response		%
Heavy metals and other trace elements		71
	Non-specific	20
	Lead	66
	Arsenic	11
	Mercury	4
	Chromium	4
	Cadmium	3
	Copper	1
Organic chemicals		36
	Petrochemicals (e.g., fuel, oil)	19
	Pesticides	13
	Persistent organic pollutants	7
	Automotive fluids	6
Chemicals (non-specific)		16
Biological hazards		11
	Human excreta	7
	Animal excreta	6
Building materials (e.g., asbestos, asphalt, roofing tar)		11
Foreign objects (e.g., trash, needles)		21
Other		9
No response		10

Gardeners were also asked to list any health concerns they have as community gardeners (Table 2); half (51%) cited soil contaminants as among their concerns. When asked to express their overall levels of concern about contaminants in their gardens, the average response was 2.3 on a scale of one to five, with five being the most concerned. There was no apparent association between levels of concern and whether gardeners thought their soil had been tested, or what they thought the test results indicated (Table 3).

**Table 2:** Open-ended responses to “As a community gardener, do you have any concerns about hazards to your health?”

Response		%
Soil contaminants		51
	Non-specific	20
	Organic chemicals (e.g., pesticides)	16
	Heavy metals and other trace elements	11
	Trash	11
	Discarded needles	9
	Human or animal excreta	4
Crime		6
Animal pests		4
Injury		3
Air quality		3
No concerns		44

**Table 3:** Gardeners' levels of concern about contaminants in their garden, by perceived soil test status.

“Has your soil been tested for contaminants?”	“Did the results reveal a problem with contamination?”	Number of respondents	Average level of concern (1–5)* among respondents
No	NA	14	2.5
Unsure	NA	16	1.7
Yes	No	20	2.6
Yes	Unsure	9	2.0
Yes	Yes**	11	2.4

NA: Not applicable.

* On a scale of one to five, with five being the most concerned.

** 8 of these 11 respondents indicated they discontinued growing food crops in contaminated areas; two indicated the soil was remediated; one was unsure whether corrective action was taken.

When asked to list the ways in which one might come into contact with contaminants, most (70%) surveyed gardeners mentioned ingestion (e.g., “eating crops”). They did not specifically mention incidental ingestion, e.g., accidentally swallowing small amounts of soil while gardening. Other responses included dermal contact with (63%), and inhalation of (39%) contaminants.

Through interviews it became clear that lead is the contaminant of greatest concern among informants and, based on informants' perceptions, also the most common contaminant concern among gardeners. Informants expressed particular concerns about the vulnerability of children to contaminants—and specifically to lead—as compared to adults; among gardens where children may be present, some informants emphasized the heightened importance of testing their soil and making sure children do not ingest it. Informants were also concerned about other contaminants such as trash, drug paraphernalia, and animal feces, as well as potential contaminants in fill dirt, compost, and water. Informants also expressed concerns about chemical inputs, such as pesticides, and indicated that gardeners may view their use as more harmful to health than contaminants directly in soil. Table S3 in File S1 illustrates the range of concerns noted in these discussions.

Most informants did not express a high level of concern about soil contaminants. Frequently, informants indicated that their concerns about soil contamination were alleviated by the use of raised beds or after seeing safe results come back from soil tests. Informants also made repeated comments about soil quality, suggesting issues of soil fertility may be more salient than contaminant concerns.

*For me, the most important thing is that we have good soil structure here. … So, really my energies have been not around contaminations, but just building healthy soil so that we get the best vegetable production out of here. (Community garden leader)*

In discussing gardeners' levels of awareness or concern about soil contamination, informants' views varied widely. Several had little confidence that gardeners think about soil contamination as an issue or are aware that soil testing can and should be conducted. Informants working with municipal programs related to community gardening noted that few people ask about soil contamination when starting a garden. At the other end of the spectrum, some informants noted broad concern about soil contamination among community gardeners.

*We talk about air. We talk about water. … Nobody talks about soil, and, essentially, it became very obvious that soil's probably the most contaminated thing that we have in our environment, particularly in urban areas.* (Master Gardener)

*I think each year, the new gardeners ask do we have to be concerned about soil, and I say we tested it and everything was okay.* (Community garden leader)

*And I've noticed that most gardens want raised beds, because they think there is a lead issue. (City government representative)*

Informants noted that knowledge and concerns about contaminants vary with different populations. Younger and more educated individuals, for example, were thought to have greater awareness of soil contaminant issues. A few informants noted that soil contamination was of less concern for gardeners and volunteers at urban farms, because they trusted that the appropriate steps had been taken to ensure the safety of the soil. One urban farmer noted that community members were concerned about soil contamination when the farm was first getting started, but once it became “established”, these concerns disappeared.

### Site history

One of the first steps in determining soil safety is learning how a particular site was used. This was a top concern of one City government representative, who worried that by testing soil without the knowledge of a site's history, gardeners may be unaware of potential contaminants:

*If you do a test for lead and other heavy metals and you pat yourself on the back and you go on, are you missing the fact that there used to be a gas station on that site and there could be types of contaminants that you don't even know how to test for that could pose a risk?*

Most (73%) surveyed gardeners said they knew the site history of their gardens. Likewise, the community garden leaders and urban farmers we interviewed indicated they knew the past use of their garden and farm sites.

When asked how they learned about their site's history, most community garden leaders and urban farmers we interviewed indicated they spoke to residents in the surrounding area. One urban farmer said they would start by referencing Sanborn Maps, which are available through local libraries and depict historical land uses from 1867 to 1970. Surveyed gardeners reported obtaining information on site histories primarily from other gardeners (42%), from neighbors (26%) or based on their own observations (23%). A small proportion (7%) had obtained information from a government office, such as the Department of Planning.

When asked if site history is important information in determining if a site is suitable for gardening, nearly all (99%) surveyed gardeners agreed. In contrast, one City government representative suggested that most gardeners would not be interested in trying to uncover information on site history, noting, “I think, based on the people I've met and talked to, they just wanna grow something.”

Informants also suggested that gardeners may lack the expertise necessary to conduct a site history. One City government representative noted that in an ideal world, assistance would be provided to new gardens to test the soil and “sit down with somebody and go over the site history in a way that's simple and doesn't take too long and is very clear.” One federal agency employee indicated that expert guidance is the “crucial part” of a Phase 1 Environmental Site Assessment, in which an auditor reviews historical and other records, visits the site, and interviews previous landowners and other informants:

*[T]he site history will get you … maybe 85% there.... [T]here's nothing about [a Phase 1 assessment], less judgment, that a person can't do at the library. But that is still missing that crucial part of the equation—the expertise piece.... Because you'll see maybe it was a paint factory. Well, will everybody think about, “Okay, well, how was a paint made at that time”? Well, it was linseed oil and the white lead was actually mixed in, and it was about at 40% lead.*

Another federal agency employee described additional limitations of site histories, noting that “the use and dumping on backyards and gardens is so idiosyncratic that it's impossible to learn what happened even ten years before,” and that conducting sporadic site histories on a per-garden basis may not reveal evidence of contaminants from other parts of the city (e.g., from nearby industries that have since shut down). For these reasons, this informant recommended a more comprehensive approach of conducting site histories and soil testing at a city-wide level.

### Soil testing

One theme that emerged during informant interviews was the barriers that deter gardeners from testing their soil for contaminants.

Cost was perceived to be prohibitive, particularly in situations in which a gardener wanted to test for a contaminant outside the scope of the usual metals panel, such as asbestos. According to informants, these situations also required additional knowledge about what to test for and how to find a service that offered those tests.

Informants also suggested that gardeners might perceive the steps involved in taking and sending away soil samples to be cumbersome or too complicated. Some informants suggested the need for a local testing service or a government-funded public service for soil testing, although not all agreed that a local testing service would be worth the cost.

*There should be an immediate way to get testing … because some people will not go through the process of sending something away.* (Community garden leader)

*I don't really see a huge a demand out there for this kind of information … it takes a whole lot to get the right people together to have a soil-testing lab here, in the city. And then, again, it might not even be used.* (City government representative)

Other barriers mentioned by informants included fear of discovering contamination after having already made investments in the land, and the need to document the exact locations from where soil samples are taken within a garden (since contaminant levels may vary spatially across a garden).

A few informants also noted that once soil tests are conducted, gardeners might have difficulty interpreting the results. Our survey results also hinted at this – in responding to questions about tests for contaminants, several gardeners referenced nutrient levels and soil tilth, suggesting that some gardeners may conflate tests of soil fertility with tests for contaminant levels.

Several informants also perceived a lack of scientific consensus about what levels of contamination are considered safe.

*[H]ow much lead is too much lead? I have read different numbers. In Canada, the safe level is different than in the United States, and I think that in Europe it's different.* (City government representative)

*[T]here's a lot of conflicting information from EPA and different universities about what an action level would be for total lead...* (Agricultural extension employee)

Both federal agency employees – experts in contamination – also noted imperfections in the science in determining risk standards.

### Reducing exposure to contaminants

Another aim of this study was to explore knowledge and practices related to reducing exposure when working in potentially contaminated gardens.

We asked surveyed gardeners how they thought one should approach working in contaminated environments, and what they would do if their soil was found to be contaminated; these results are summarized in Table 4.

**Table 4:** Open-ended responses to questions about reducing exposure in contaminated environments.

Response		%
Stop growing produce in contaminated areas, and/or stop eating produce grown in contaminated areas		50
Remove surface contaminants		26
	Wash produce	26
	Peel root crops	3
Remediate soil		26
	Install a barrier over contaminated soil	9
	Add soil amendments (e.g., compost or minerals)	9
	Bioremediate, phytoremediate, and/or mycoremediate	9
	Remove contaminated soil	9
	Remediate (non-specific)	4
Grow in raised beds or containers		17
Only grow certain crops (e.g., not root vegetables)		13
Wear gloves		9
Wash hands		6
Apply mulch (e.g., to reduce splashing on crops)		3
Continue using the same methods		3
Seek out more information		29
Don't know		24

“What methods should one use to grow, harvest or handle produce grown in contaminated environments?” and “What would you do if you found out your soil was contaminated?”

Among interviewed informants, most cited building raised beds and filling them with clean, imported soil as the safest and easiest way to manage potential soil contamination. Among surveyed gardeners, using raised beds was a common practice. The majority (74%) reported growing at least some crops in raised beds, and 50% said they use raised beds exclusively. Some informants, however, alluded to concerns regarding limitations of raised beds. One federal agency employee noted the possibility that the soil used to fill raised beds may be contaminated, particularly if it was taken from a questionable source, and that plant roots may extend down into contaminated soils below the raised bed, potentially allowing contaminants to enter plant tissues. One urban farmer also suggested that people may be exposed to contaminants from soil not covered by the raised bed (e.g., if native soil is kicked up), and that gardeners who use raised beds might underestimate these risks and not test the underlying soil.

We also asked informants and surveyed gardeners about soil remediation. One City government representative suggested that gardeners may not necessarily know how to remediate the soil and that guidance is needed to provide direction. When surveyed gardeners were asked, for example, whether they thought planting sunflowers in contaminated soil would effectively remediate it, 9% incorrectly said yes and 51% were unsure.

### Information sources

Another aim of this study was to understand where gardeners obtain information about soil contamination. Surveyed gardeners (see Table 5) and interviewed informants most commonly mentioned gardening support institutions, particularly the agricultural extension office and its Master Gardener program.

**Table 5:** Open-ended responses to “Where do you get information on gardening practices?”

Response		%
Gardening support organizations		84
	Extension office/Master Gardeners	37
	CGRN	19
	Other	4
Online		53
Other gardeners		44
Books/magazines		29
Friends/family		16

One theme that emerged from informant interviews was the need for a central repository where gardeners could access information about soil contamination. Most informants thought this should be offered through an organization that gardeners already associate with gardening information. Compared to the more formal services of the agricultural extension, the community-based CGRN was cited as having “the biggest direct communication with community gardeners in the city” and being “more accessible” than more “bureaucratic” organizations. One informant noted, however, that while it provides a valuable network for gardeners, within CGRN “a lot of misinformation gets shared.” The agricultural extension was thought to be the traditional place where gardeners and farmers would think to access soil contamination information, but informants noted it had “not historically had a real urban presence” and was not prepared to deal with issues common in cities, such as urban soil contamination. One City government representative saw the municipal government as best positioned to gather, hold, and disseminate information on soil contamination:

*[G]overnment can play a really important role in doing the due diligence and gathering all of that stuff and then making it publicly available. … I think we have the institutional longevity to hold on to that [information]. … And we need to have this historical data so if that [soil] test has already been done you don't need to go do it again.*

### Information needs

Through informant interviews, we aimed to identify urban community gardeners' information needs related to soil contamination. Responses fell under four main topics: site history, soil testing, remediation, and minimizing exposure (see Table 6).

**Table 6:** Community gardeners' information needs related to soil contamination, as reported by key informants.

Site history	How to find information about past uses of a plot of land
	Which contaminants to test for, given specific past land uses
	Geographic areas of the city where there are likely to be high levels of contamination
Soil testing	Importance of obtaining a soil test prior to gardening
	Which contaminants to test for
	Why to test for certain contaminants and not others
	Where to get soil testing done
	How much soil testing costs
	How to correctly take a soil sample for a soil test
Remediation	Best practices for remediating contaminated urban soils
Minimizing exposure	How to reduce exposure risks when gardening
	Contamination risks associated with imported materials such as compost or mulch

Regarding how best to present this information to gardeners, the overriding theme was that it must be concise, clearly organized, and use simple, straightforward language that speaks to people of varying educational levels. One City government representative noted that the information that is needed already exists, but should be combined “in a document with clear instructions that a layperson would feel comfortable using.”

One community gardening organization representative also placed value in communicating the “fluid” nature of determining what levels of contaminants are considered safe, and that the “best way … [to approaching contaminant issues is] not black and white.” Furthermore, informants emphasized the need to balance risk reduction messages related to soil contamination with the health, social, and environmental benefits of gardening, as well as “the values in gardening that are beyond measure.”

*[W]e don't want to create barriers. We want more people to be growing food. (City government representative)*

### Disseminating information

Another consideration is how best to disseminate information about soil contamination to community gardeners. Informants had a broad range of suggestions, and several noted that a combination of dissemination strategies – including print, online, and face-to-face information – is needed in order to reach all types of gardeners.

Most informants expressed a need for more interactive, face-to-face methods of dissemination, such as individual consultations, workshops, or a citywide gardening conference. The important role of Master Gardeners was noted, for, as stated by one City government representative, they are “already tapped into the lives of the gardeners out there in the world.”

*[N]o matter how hard we try we may never reach half the people who want to garden. Because they're not going to ask, they're not going to look, they're not going to read, they're not going to do a Web search. … So only by person-to-person communication pushed out from the community master gardeners. (Federal agency employee)*

### Training needs

While we did not ask informants about their level of knowledge related to soil contamination, many spontaneously acknowledged confusion and a lack of understanding about the issue. One federal agency employee recommended that to help urban community gardeners make decisions about the safety of garden sites, training is needed to provide plant and soil experts with expertise on soil contamination.

*[T]he only really education I've had about soil is what we had in our Master Gardener class. And to be honest, that was very cursory, tip of the iceberg, basic soil composition. And I don't really remember … there being a lot of information shared about contamination. (Community garden leader)*

## Discussion

Through surveys with urban community gardeners and key informant interviews, we characterized urban community gardeners' knowledge and perceptions of soil contaminant risks, including their knowledge of how to assess and reduce these risks, sources of information on soil contamination, and information needs related to soil contamination.

### Knowledge and concerns

Our results suggest that concern about soil contaminants among community gardeners in Baltimore is generally low, particularly among established gardens. Informants indicated this is likely because gardeners assume soil contamination has already been addressed through safe soil test results, remediation, or the use of raised beds. Concern may be warranted, however, since prior studies of Baltimore soils suggest that soil contaminant levels vary widely [24]–[26]—even within the same garden plot [25]—and at some sites lead levels greatly exceed EPA screening levels (Table S1 in File S1). Soil lead levels have also been shown to increase over time due to atmospheric deposition [37]. Informants called for extra precautions where children may be present in gardens, echoing evidence of children's vulnerability to soil contaminants [24], [29].

Surveyed gardeners' knowledge and concerns related to soil contaminants largely focused around lead. Their awareness may have been informed by recent state and city interventions in Baltimore aimed at raising awareness of child lead poisoning [38], [39]. Gardeners' concerns were in accordance with tests of Baltimore soils (Table S1 in File S1), which identified high levels of lead more often than other trace elements included in analyses. Gardeners demonstrated inconsistent awareness about other potential contaminants.

Among key informants, issues that affect gardeners' ability to cultivate plants often appeared to be more salient than contamination concerns. Additionally, our results suggest that Baltimore's gardeners are more concerned about chemicals added to the gardening environment than what contaminants may already be present in soil. Gardeners' concerns about pesticides reflect the results of prior surveys that found the primary reason consumers purchase organic produce is to reduce exposure to pesticides and other chemicals [40].

Our results identified areas where gardeners' knowledge and concerns may not be concomitant with the potential health risks associated with urban soil contaminants. Efforts to address discrepancies in gardeners' knowledge, however, must be carefully crafted so as to not elevate levels of concern beyond those at which they would discontinue gardening altogether. Informants also made the important point that risk reduction messages must be balanced with the health, social, and environmental benefits of gardening.

### Site history

Our results suggest gardeners recognize the importance of knowing a garden site's prior uses. Several informants indicated, however, that gardeners may lack the motivation, information and expertise to determine accurately the prior use of their garden site, or to anticipate the contaminants that may be present as a result. City-wide documentation of site histories may be an effective means to alleviate these responsibilities from gardeners.

### Soil testing

Our findings also revealed potential barriers to soil testing, including not knowing how to properly sample soil from a garden, where to send soil samples for testing, and which contaminants to test for under various circumstances, as well as the perception that testing is too expensive, complicated, or cumbersome. Even when soil tests are conducted, gardeners may have difficulty interpreting the results. Given surveyed gardeners' knowledge and concerns were largely centered around lead, they may interpret a negative test result for lead as a “clean bill of health” and neglect to consider the presence of other contaminants. These and other concerns speak to the potential value of providing gardeners with cost-assistance and guidance on testing soil and interpreting results. Such services could, for example, be included as part of a lease to adopt city-owned vacant lots.

### Reducing exposure to contaminants

When surveyed gardeners were asked to list practices to reduce exposure in contaminated environments (Table 4), several best management practices [25], [41], [42] were notably absent from responses, including reducing soil ingestion among children (e.g., by washing their hands, and reducing hand-to-mouth contact), growing produce away from busy streets, railways, and older buildings, and removing shoes to avoid tracking contaminants into the home. Few respondents alluded to the use of mulch (3%) to reduce splashing on crops; or the removal of surface contaminants, e.g., by washing produce (26%) and peeling root crops (3%). Other gaps in practices included the application of soil amendments (9%) to dilute contaminants and/or reduce their mobility or bioavailability (e.g., applying phosphorus to reduce the bioavailability of lead [29]), though such amendments may also *increase* the mobility or bioavailability of certain contaminants [43]. Some gardeners were quick to acknowledge their limited knowledge on how to approach contaminated environments, and indicated they would seek out more information (29%) or take the conservative approach of not growing produce in and/or eating produce from contaminated areas (50%).

Contaminant concerns among gardeners and informants were often alleviated by the use of raised beds, which were viewed as an easy and effective solution to managing soil contamination. As some informants noted, raised beds do not remedy the presence of contaminated soil surrounding the bed, which may be kicked up during gardening activities. Clark and colleagues [37] raise particular concerns regarding children: based on a model specific to lead-contaminated gardens that considered incidental soil ingestion, inhalation of ambient air, and consumption of tap water and garden-grown produce, an estimated 72–91% of children's lead exposure is via incidental soil ingestion. Because raised beds only cover a small percentage of land, they offer limited protection against incidental ingestion via hand-to-mouth behavior among children playing in areas with contaminated soils. Raised bed soils may also become contaminated over time; lead levels measured in urban raised beds in Dorchester, Massachusetts were found to increase by roughly 185 parts per million over a four year period due to wind-transported fine grain soil [37]. Another study explored concerns related to the use of timbers in the construction of raised beds, detecting elevated levels of arsenic in garden plots framed by chromated copper arsenate treated lumber and elevated levels of polycyclic aromatic hydrocarbons in plots framed by railroad ties [44]. In addition to these concerns, as one informants noted, soil used to fill raised beds may be contaminated, and plant roots may extend into contaminated soils below the bed. While raised beds provide some protection against contaminant exposure, they are not a panacea, and recommendations for their use should be tempered with information about their limitations.

### Information and training needs

Informants voiced a need for specific information related to the management of soil contamination and indicated that gardeners need a central place where they can access such information. They noted several potential organizations that could serve such a role. Informants suggested collaboration between such organizations and City government to develop and disseminate a single set of information could yield the greatest reach.

Informant interviews suggest the major challenges in providing such information are the complexity and uncertainty surrounding the issue. Thus, there may be a role for two levels of guidance: additional training for gardening experts, to help them better understand the issues around soil contamination and how to effectively communicate risks to community gardeners; and concise, straightforward messaging for gardeners.

### Study limitations

Our sample population was small, and the median income bracket among surveyed gardeners ($50,000 – 60,000) and percentage with a bachelor's degree (83%) were high relative to the population of Baltimore City in 2007–2011 [45]. For these reasons, our study population may not be representative of the average Baltimore gardener. Our qualitative findings may also be unique to the Baltimore context; however, given the lack of research on this topic, we believe the results of this study can be used as a starting point to inform educational interventions for reducing soil contamination risks among gardeners in a variety of urban contexts.

## Conclusions

Through this study, we identified a range of factors, challenges, and needs related to Baltimore community gardeners' perceptions of risk related to soil contamination, including low levels of concern and inconsistent levels of knowledge about heavy metal and organic chemical contaminants, barriers to investigating a garden site's history and conducting soil tests, limited knowledge of best practices for reducing exposure, and a need for clear and concise information on how best to prevent and manage soil contamination. Key informants discussed various strategies for developing and disseminating educational material to gardeners. For some challenges, such as barriers to conducting site history and soil tests, some informants recommended city-wide interventions that bypass the need for gardener knowledge altogether. In cases where public health messages about the risks from soil contaminants are implemented, informants stressed the importance of crafting messages in ways that do not dissuade gardeners from continuing to garden in urban environments. Given the health, social, environmental, and economic benefits associated with participating in and supporting urban green spaces, it is critical to protect the viability of urban community gardens while also ensuring a safe gardening environment.

## Supporting Information

File S1 **Supporting Information Tables. Table S1, Levels of selected heavy metals and other trace elements detected in Baltimore soils.** Heavy metals panels do not detect for the range of contaminants that may be present in soil (e.g., organic chemicals), and should be paired with information on a site's prior use before proceeding with gardening activities. Reported levels may be capped at minimum and maximum detectable levels. **Table S2, Examples of potential urban soil contaminants, recognized sources, and health effects associated with exposure. Table S3, Examples of contaminant concerns expressed by key informants.**
(DOCX)
