# Supplementary material for: Urban Community Gardeners' Knowledge and Perceptions of Soil Contaminant Risks
Source: PLoS One. 2014 Feb 6;9(2):e87913. doi: 10.1371/journal.pone.0087913 (PMC3916346; doi:10.1371/journal.pone.0087913)
Supplement: File S1 — Supporting Information Tables. Table S1, Levels of selected heavy metals and other trace elements detected in Baltimore soils. Heavy metals panels do not detect for the range of contaminants that may be present in soil (e.g., organic chemicals), and should be paired with information on a site's prior use before proceeding with gardening activities. Reported levels may be capped at minimum and maximum detectable levels. Table S2, Examples of potential urban soil contaminants, recognized sources, and health effects associated with exposure. Table S3, Examples of contaminant concerns expressed by key informants. (DOCX) [file pone.0087913.s001.docx]

**Table S1. Levels of selected heavy metals and other trace elements detected in Baltimore soils.**

Heavy metals panels do not detect for the range of contaminants that may be present in soil (e.g., organic chemicals), and should be paired with information on a site’s prior use before proceeding with gardening activities. Reported levels may be capped at minimum and maximum detectable levels.

|  |  | **N** | **Arsenic** | | **Cadmium** | | **Chromium** | | **Copper** | | **Lead** | | **Manganese** | | **Nickel** | | **Zinc** | |
| --- | --- | --- | --- | --- | --- | --- | --- | --- | --- | --- | --- | --- | --- | --- | --- | --- | --- | --- |
| **Study** | **Sampled land** |  | **Median** | **Max.** | **Median** | **Max.** | **Median** | **Max.** | **Median** | **Max.** | **Median** | **Max.** | **Median** | **Max.** | **Median** | **Max.** | **Median** | **Max.** |
| Mielke et al. (1983) | Vegetable gardens | 422 |  |  | 0.56 | 13.65 |  |  | 17 | 97 | 100 | 10,900 |  |  | 3 | 53 | 92 | 4,880 |
| Chaney et al. (1984) | Vegetable gardens | 50 |  |  | 1.72 | 13.60 |  |  | 54 | 293 | 573 | 10,900 | 140 | 386 | 5 | 25 | 352 | 4,880 |
| Pouyat et al. (2007) | Various* | 122 |  |  | 0.89 | 3.10 | 38 | 794 | 35 | 216 | 89 | 5,652 | 422 | 2,125 | 18 | 336 | 81 | 1,109 |
| Schwarz et al. (2012) | Residential | 1,121 |  |  |  |  |  |  |  |  | 124 | 9,151 |  |  |  |  |  |  |
| Current study** | Vegetable gardens | 13 | <1.0 | 3.0 | 0.41 | 1.74 |  |  | 22 | 65 | 67 | 495 | 210 | 522 | 2 | 4 | 72 | 157 |
| SSL***, carcinogenic | | | 0.39 | | 1,800 | | 0.29 | | N/A | | N/A | | N/A | | N/A | | N/A | |
| SSL, non-carcinogenic | | | 22 | | 70 | | 230 | | 3,100 | | 400 | | 1,800 | | 3,700 | | 23,000 | |

*Residential, forest, park, institutional, commercial, transportation, and open urban land.

**Two of the 15 participating gardens were enrolled after soil samples were sent out for analysis.

***Soil Screening Level, as determined by the U.S. Environmental Protection Agency. Sites containing contaminants at levels above SSLs may require further evaluation, but are not necessarily unsafe for gardening, particularly if children are not active in the garden and steps are taken to minimize exposure to contaminants. See U.S. Environmental Protection Agency (2012) Regional Screening Level (RSL) Resident Soil Table, available at [www.epa.gov/region9/superfund/prg/](http://www.epa.gov/region9/superfund/prg/).

**Table S2. Examples of potential urban soil contaminants, recognized sources, and health effects associated with exposure.**

| **Contaminant** | **Sources** | **Health effects** |
| --- | --- | --- |
| Lead | Burning coal, lead-acid batteries, leaded gasoline, lead-based paints, solder | Neurological impacts, encephalopathy, bone deterioration, hypertension |
| Arsenic | Certain pesticides, treated lumber, burning coal | Gastrointestinal damage, skin damage, cancer, neurologic impacts, heart and liver damage |
| Cadmium | Burning coal, batteries, phosphate fertilizer, galvanized water pipes | Liver and kidney damage, cancer, decreased bone density |
| Chromium | Metal plating, treated lumber | Cancer, gastrointestinal disorders, hemorrhagic diathesis, convulsions |
| Organic chemicals | Fuels, oils, pesticides, plastics, lubricants, refrigerants, cleaning solvents, preservatives, flame retardants | Varies by chemical; may include cancer and disruption of reproductive, immune, endocrine and nervous systems |
| Asbestos | Attic and wall insulation, insulated water pipes, roofing shingles, ceiling and floor tiles, cement, automobile parts | Lung cancer, mesothelioma, and other respiratory diseases |
| Biohazards | Untreated human and animal waste, septic tanks, municipal waste water | Bacterial, viral, or parasitic infections |

Adapted from Brevik EC (2013) Soils and human health: an overview. In: Brevik EC, Burgess LC, editors. Soils and human health. CRC Press. pp. 29–56.

**Table S3.** **Examples of contaminant concerns expressed by key informants.**

| **Concern** | **Exemplary quotes** |
| --- | --- |
| Lead | *Community gardens are much more likely to have kids in them, which is really a sensitive population for lead. So we're very concerned about getting the word out, at the very least, about testing your garden for lead, or growing raised beds for community gardeners for the safety of kids.* (City government representative) |
| Pesticides | *I hear people defending the food that they’re growing a lot and comparing it to what you might purchase in a grocery … And they talk about chemicals and pesticides and preservatives and what’s safe. We have had that conversation about safety [related to soil contamination] a lot, but I think that [the use of chemical inputs] really is in a gardener’s mind… this fate that they are creating something that’s healthier or better for themselves regardless of those potential [soil contamination] risks.* (Community gardening organization representative) |
| Chemical fertilizers | *I figure … that Miracle Grow and all these other chemicals, especially the farmers and the stuff they use, that's what's giving you cancer. That's what's killing you … because if you can make a plant grow, get big size like that, what does it do when you eat it? So I just don't fool with it.* (Community garden leader) |
| Trash | *[W]e've gotten [the neighborhood kids] excited about eating food. … They'll eat the tomatoes right off the plants … [T]hat's another reason the trash has been a major issue for me is just, if it's just floating around everywhere and it's just getting right into crops, it makes me feel like I can't just eat the tomato right off the plant. … And that's what you should be able to do on an organically grown plot.* (Urban farmer) |
| Drug paraphernalia | *A hazard that I think that is… recently in my head because I noticed how comfortable I got with it are really just needles in the garden and I guess other kinds of drug-related paraphernalia… When people are cleaning up a lot of the vacant lots, we're finding lots of those things … that are going to go through your gloves...* (Community gardening organization representative) |
| Animal feces | *[T]hey were vacant lots that people had used as dog runs. And they were gross. So, even though we raked as much of that stuff out of there as possible, the thought of growing beets in that was just disgusting*… (City government representative) |
| Contaminated water | *I worry about the water sometimes, the water coming out of the hydrants. Sometimes it's brown. So I get nervous about that. Like, oh, should I be testing this water?* (Urban farmer) |
| Contaminated compost | *The question [gardeners] ask me the most though, when we were using the composted leaves was about whether that was tested. … [T]he perception is because these leaves are scraped up off of the street, the big sweepers go through and you would find broken bottles, old tires, parts of a curb, all kinds of things would show up in this leaf mold. And so, people became very concerned about what was in it…* (City government representative) |
| Contaminated fill dirt | *I mean, that's where the real level of concern should be really is, well, where did that [fill] dirt come from? Does the city have regulations?* (Urban farmer) |
